# Niche Differentiation at Multiple Spatial Scales on Large and Small Mediterranean Islands for the Endemic *Silene velutina* Pourr. ex Loisel. (Caryophyllaceae)

**DOI:** 10.3390/plants10112298

**Published:** 2021-10-26

**Authors:** Valentina Murru, Emmanuele Farris, Andrea Santo, Oscar Grillo, Carole Piazza, Antonella Gaio, Gianluigi Bacchetta, John D. Thompson

**Affiliations:** 1 Centro Conservazione Biodiversità (CCB), Dipartimento di Scienze della Vita e dell’Ambiente (DISVA), Università degli Studi di Cagliari, V.le S. Ignazio da Laconi 13, 09123 Cagliari, Italy; muva@tiscali.it (V.M.); andrea.santo85@gmail.com (A.S.); oscar.grillo.mail@gmail.com (O.G.); bacchet@unica.it (G.B.); 2 Dipartimento di Chimica e Farmacia, Università degli Studi di Sassari, Via Piandanna, 4-07100 Sassari, Italy; 3 Conservatoire Botanique National de Corse, Avenue Jean Nicoli, 20250 Corte, France; Carole.Piazza@oec.fr; 4 Parco Nazionale Arcipelago di La Maddalena, Via Giulio Cesare, 7-07024 La Maddalena, Italy; a.gaio@lamaddalenapark.it; 5 Hortus Botanicus Karalitanus (HBK), Università degli Studi di Cagliari, V.le S. Ignazio da Laconi 9-11, 09123 Cagliari, Italy; 6 Centre d’Ecologie Fonctionelle et Evolutive, UMR 5175, CNRS, EPHE, IRD, Université Paul Valéry Montpellier 3, Route de Mende 1919, 34293 Montpellier, France; john.thompson@cefe.cnrs.fr

**Keywords:** demographic strategies, ecological release, endemism, niche variation, persistence niche, plant population dynamics, small island effect (SIE)

## Abstract

The aim of this work is to investigate niche variations in endemic *Silene velutina* (Caryophyllaceae, Angiosperms) on Mediterranean islands that differ in size. Six populations on both large and small islands were sampled across the geographic range of the species. For each population, 10 plots (1 × 2 m, with a 25 cm grill) were randomly placed to quantify environmental (abiotic and biotic factors and disturbance) and population (demographic structure and reproductive success) parameters. Niche parameters related to substrate, plant cover, community diversity and composition and disturbance showed significant variation in relation to island size. At the regional scale, we detected a broader niche on large islands associated with spatial heterogeneity and island size. In contrast, at the local scale, populations on small islands showed a broader niche, potentially due to a release from competition (low diversity and plant cover and absence of phanerophytes). Populations on large islands had a demographic structure biased towards vegetative individuals (seedlings and juveniles) with few reproductive individuals, while those on small islands had a majority of adults. Together, the results on niche breadth and demographic structure concord with the idea of a strategy based on adult persistence on small islands.

## 1. Introduction

Island archipelagos have long fascinated biologists because of the originality of their fauna and flora [1,2]. As a result of their isolation, patterns and processes of biodiversity evolution often differ on islands compared to mainland situations, as observed for population and community dynamics, species composition and species richness [3,4,5,6].

The rate at which species increase in number with the augmentation of island surface area is a fundamental issue in island ecology and biogeography [3,7]. A major correlate of increasing species diversity with island size is habitat diversity [8,9,10,11,12,13]. In fact, an increase in island size is often associated with increasing ecological complexity, which takes the form of a landscape mosaic of different environments. In addition, since the formulation of the Theory of Island Biogeography and the so-called “species–area relationship” on islands [3], anomalous patterns for this relationship have been detected on small islands, the so-called “small island effect” [14,15,16,17]. In addition, species diversity can be modified by disturbance, which can alter the environmental conditions and relationships among individuals, populations and species, by producing new open areas and reduced competition [18,19].

The Mediterranean Basin, with its recurrent juxtaposition of large islands and more than 1000 minor islands in the western Mediterranean Sea [20], represents an ideal natural laboratory for studies of insular ecology and biogeography. Most Mediterranean islands are once-connected continental fragments or land-bridge islands [21] that were historically linked to adjacent continents (“continental islands” sensu [22]). On these islands, species richness is often significantly correlated with both area and elevation, but poorly with distance from continental areas, because their floras were very similar prior to isolation [21,23,24,25,26]. Of major importance for species diversity on large Mediterranean islands is thus the elevation gradient and habitat diversity [21].

Vascular plants can respond to environmental changes with different demographic strategies [27,28]. Two alternative strategies may evolve in habitats that differ in terms of stability, stress gradients and inter-specific competitive interactions [27,29,30,31]. A strategy of persistence is often observed in stable habitats with the in situ maintenance of established, adult plants. In contrast, a preponderant role of regeneration and the turnover and replacement of individuals by seedlings are favored in more dynamic successional or disturbed habitats.

These different demographic strategies may also reflect variation in the ecological niche. In the Mediterranean flora, endemic species have a distinct ecology compared to widespread congeners. The main element of their niche concerns their occurrence in rocky habitats on steep slopes in low, open vegetation with low species richness [21,32,33,34]. A characteristic of these habitats that may be crucial for the persistence of endemic species is that they are relatively stable, both in relation to vegetation succession and human activities. It is thus probable that the persistence of endemics may have been favored by their persistence in rocky habitats where competitive interactions are limited. In addition, Lavergne et al. [34,35] reported a clear trend for endemic species to be smaller in size and produce fewer seeds than widespread congeners and evidence that individual populations of endemic species have a greater temporal stability than that of widespread species. Populations of endemic species thus have traits, an ecological niche and recent history that all suggest high persistence and low turnover [21]. However, studies that investigate the spatial scale of ecological patterns of endemic species on Mediterranean islands remain rare, even though the realized niche is highly scale dependent [28,36,37,38]. 

In this study, we quantify the ecological niche and population structure of populations of *Silene velutina* Pourr. ex Loisel., a species that is endemic to Sardinia, Corsica and several small islands around their coastline. This study has three main objectives. First, we examine whether the species shows niche differentiation among populations on large and small islands, and, if so, whether niche differences are associated with particular types of habitat variables. Second, we examine whether there are differences in niche breadth at two different spatial scales (both local and regional scales). Third, we quantify the demographic structure of populations on small and large islands to test whether small island populations show a greater reliance on persistence due to their small size and isolation.

## 2. Results

### 2.1. Multivariate Analyses

We used linear discriminant analysis (LDA) to investigate niche differentiation among populations of *Silene velutina* large and small islands and among sites on each type of island. The two groups of islands were well distinguished, with an overall percentage of correct attribution of 91.6%, and a low percentage of misleading assignments. Large islands were correctly identified at 86.7% while small islands at 96.6% both with original data and after cross validation (Table S1). Elevation, sand, trampling, alien species and rockiness were the five variables with the greatest weight in the distinction between large and small islands. In addition, we found a correct discrimination among sites of 32.7% (considering large and small islands together; data not shown), while separate analysis showed a higher correct identification of sites on large islands (58.3%) compared to small islands (25%) (Tables S2 and S3).

**Figure 1:**
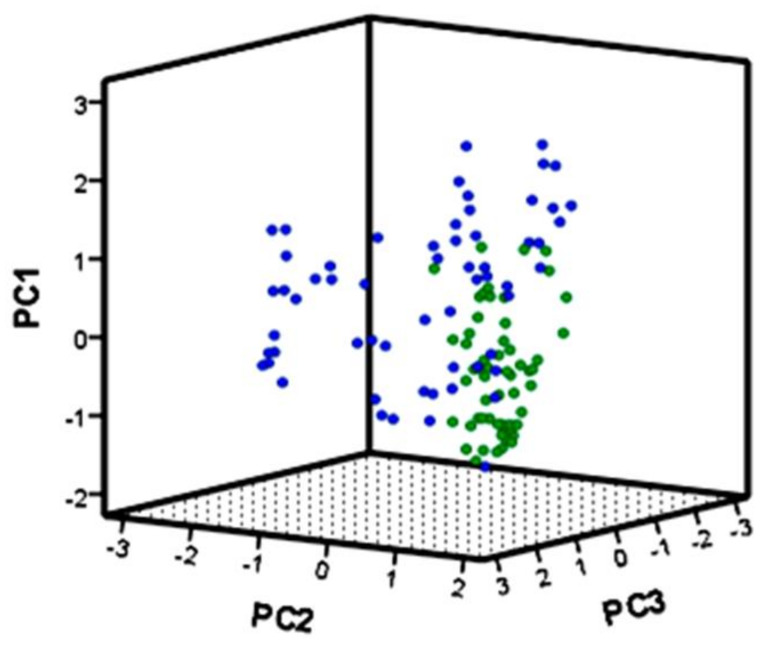
Principal component analysis (PCA) of environmental variables on three axes that explain 53% of the variation in the dataset. Dots represent plots in populations on large (blue) and small (green) Mediterranean islands.

Three principal components conjointly explained 53% of the total variance in the ecological dataset with a clear separation of large and small islands in ecological niche space (Figure 1). The first axis (PC1) was primarily influenced by biotic factors (total number of species, evenness, other species cover and total cover), the second axis was strongly influenced by substrate characteristics (slope, stoniness and sand cover) and disturbance (alien species and trampling) and the third axis was principally influenced by *S. velutina* cover and the presence of grazing (Eigenvectors of PCA are given in Table S4).

**Figure 2:**
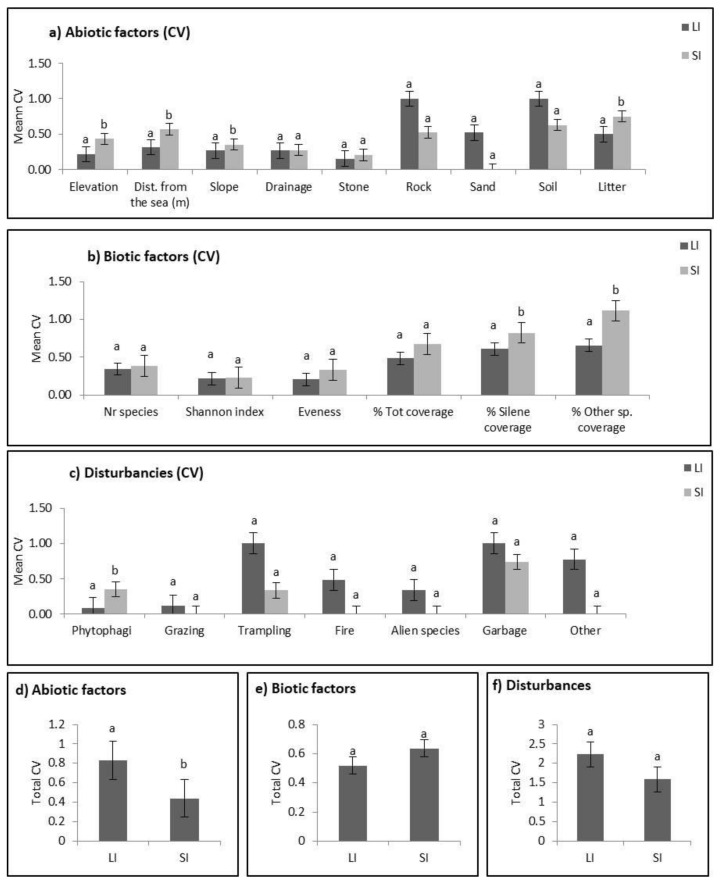
Coefficient of variation (CV) at local (**a**–**c**) and regional (**d**–**f**) scales among populations on large (LI) and small (SI) Mediterranean islands. Different lowercase letters represent significant differences at *p* < 0.05 (by SNK test).

At the local scale, mean values of the CV for abiotic (elevation, distance from the sea, slope and litter cover) and biotic (*S. velutina* cover, other species cover) variables and disturbance were higher on small islands than on large islands (Figure 2). In contrast, on a regional scale, for total abiotic factors, the mean CV values were higher on large islands.

### 2.2. Abiotic Niche Components

**Figure 3:**
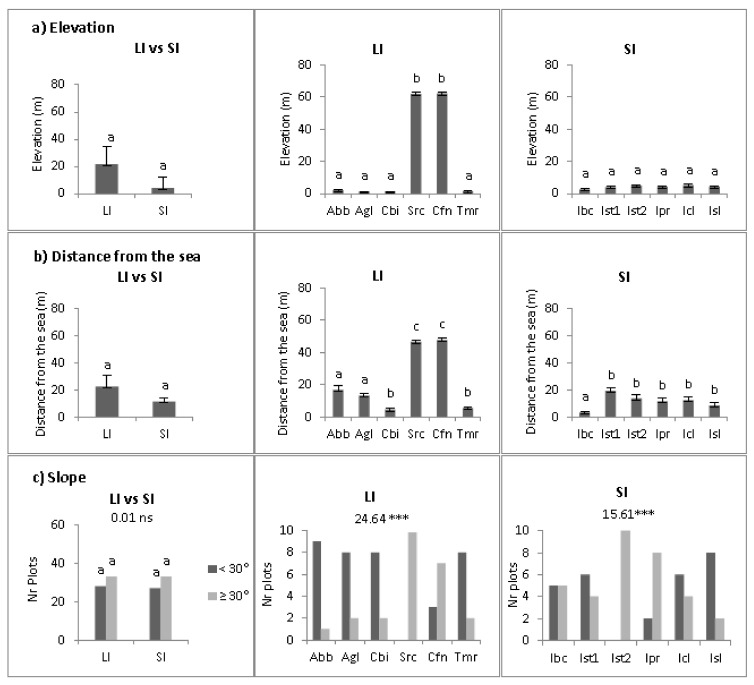
Mean elevation (**a**), distance from the sea (**b**) and slope (**c**) on large (LI) and small (SI) Mediterranean islands. A two-way ANOVA was conducted to detect differences between the two groups of islands and among sites in a and b; data are the mean of six sites (±1SE) and of ten plots (±1SE), respectively; different lowercase letters show significant differences at *p* < 0.05 (SNK test). In c a χ^2^ test with a G-test of heterogeneity were conducted to detect differences between the two groups of islands and among sites, respectively. *** *p* < 0.001.

Elevation, distance from the sea and slope were not significantly different for sites on large and small islands (Figure 3a,b). On large islands, there were significant differences (*p* < 0.05) among sites for both elevation and distance from the sea. The *Src* and *Cfn* sites were at higher elevation (ca. 60 m a.s.l.) and further from the sea (ca. 40 m) than the other sites. On small islands, significant differences among sites were found only for the distance from the sea (*p* < 0.05). Slope was highly heterogeneous among sites (*p* < 0.01) on both groups of islands. 

**Figure 4:**
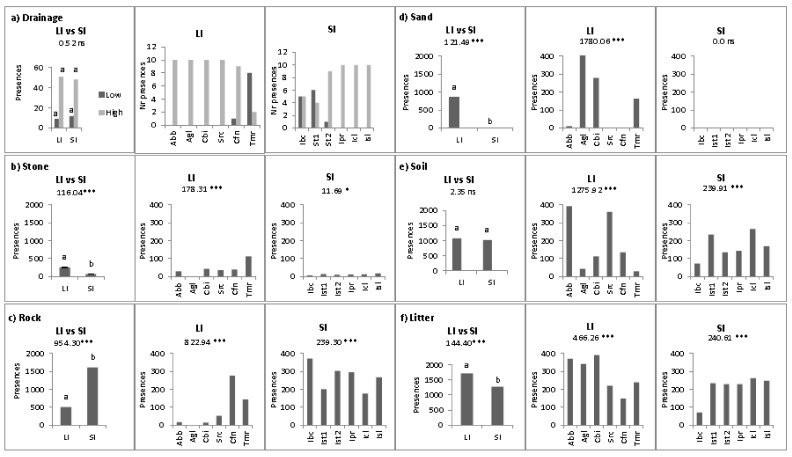
Number of point contacts (presence) with features associated with (low–high) drainage (**a**), and with stones (**b**), rock (**c**), sand (**d**), soil (**e**) and litter (**f**) on large (LI) and small (SI) Mediterranean islands, and in each site. * *p* < 0.05; *** *p* < 0.001; ns = not significant.

On both groups of islands, well-drained substrates were more common than poor-drained substrates (51 plots on LI; 48 plots on SI), with no significant differences (*p* > 0.05) in their frequency on the two groups of islands (Figure 4a). Significant heterogeneity was observed among sites both on large islands (*p* < 0.01) and small islands (*p* < 0.02), where drainage was significantly higher in five and four sites, respectively. With regard to cover variables (Figure 4b–f), on large islands there was a significantly higher (*p* < 0.01) cover of stones, sand and litter (9.7%, 31.8% and 63.4%, respectively) than on small islands (2.6%, 0%, and 47.1%, respectively) and a significantly lower cover (*p* < 0.01) of rocks (18.7% vs. 59.7%). For bare soil cover there was no significant difference (*p* > 0.05) between large and small islands (ca. 39% of soil coverage on both groups of islands). On large islands, the frequency of stones, rocks, sand, soil and litter was highly heterogeneous (*p* < 0.01) among sites (Figure 4b–f). On small islands, stone cover (*p* < 0.05) and rock, soil and litter cover (*p* < 0.01) were significantly heterogeneous among sites, and sand was absent from all monitored sites (Figure 4b–f).

### 2.3. Biotic Niche Components

**Figure 5:**
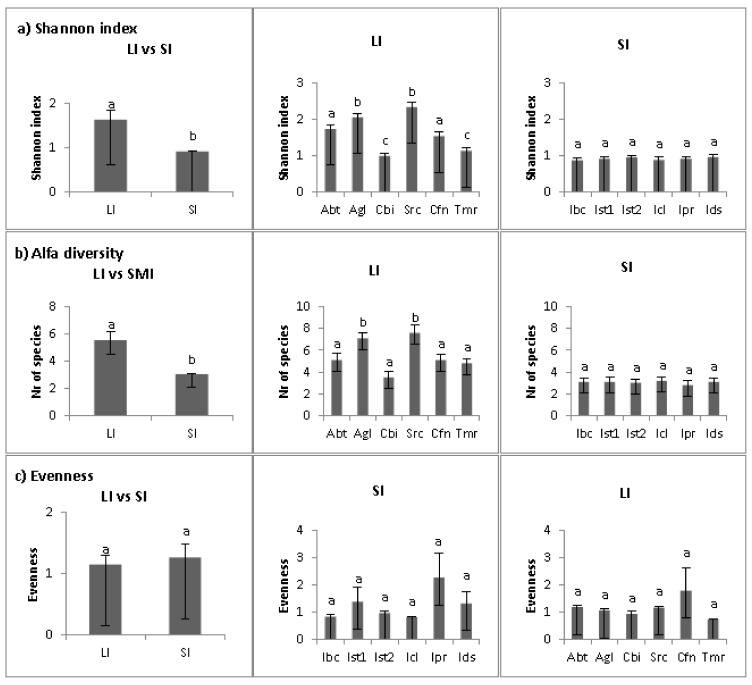
Biodiversity indexes (Shannon index (**a**), number of species (**b**) and Pielou’s Evenness index (**c**)) for populations on large (LI) and small (SI) Mediterranean islands. Different lowercase letters represent significant differences at *p* < 0.05 (by SNK test).

Overall, we found 57 vascular plant taxa on the 12 studied islands and islets (Table S5), with a maximum of 22 taxa on Src and a minimum of 7 on Ist1, Ipr and Ids. Species diversity indices were calculated per plot (2 m^2^). The Shannon index (Figure 5a) and the total number of species (Figure 5b) were significantly higher (*p* < 0.02) on large islands than on small islands, while there was no effect of island size on the Pielou’s Evenness index (*p* > 0.05, Figure 5c). For the Shannon index and the total number of species, significant differences among sites (*p* < 0.05) were found on the large islands, where the *Agl* and *Src* sites showed the highest values (respectively 7.1 ± 0.5; 7.6 ± 0.4). The Pielou’s Evenness index did not show statistically significant differences among sites both on large and on small islands. The number of species per plot was statistically higher on large islands than on small islands for phanerophytes but did not differ for other growth forms. The number of species per plot was statistically different (*p* < 0.05) among sites on large islands for therophytes, hemicryptophytes and chamaephytes but showed no significant variation on small islands.

Species frequency in one, two and three or more plots showed significantly higher values on large islands than on small islands despite the fact that, in both groups of islands, the number of species detected in three or more plots was significantly higher than that of the species detected in one or two plots. For plant cover per plot, both total cover (56,4% and 41.1% on large and small islands, respectively) and *S. velutina* cover (13.6% and 22.4% on large and small islands, respectively) were not significantly different, while other species cover was significantly higher (*p* < 0.01) on large islands (42.9%) than on small islands (17.3%). We observed highly significant differences (*p* < 0.02) among sites only for other species cover on small islands (ranging from ca. 7.6% of *Ibc* to ca. 45.3% of *Ids*). Cover per plot was significantly higher (*p* < 0.05) on large islands for phanerophytes (ca. 23.7% on LI; ca. 0.9% on SI) and showed statistically significant differences (*p* < 0.05) among sites on large islands for therophytes and on small islands for geophytes (see Figure S1 for life form cover on LI and SI).

### 2.4. Disturbance

The presence of fire and phytophagy did not differ in relation to island size (*p* > 0.05), while significantly higher levels (*p* < 0.05) of disturbance were observed on large islands for grazing (LI ca. 12%; SI ca. 0%), trampling (LI ca. 37%; SI ca. 3%), presence of alien species (LI ca. 20%; SI ca. 0%) and garbage (LI ca. 25%; SI ca. 7%). Significant differences (*p* > 0.05) were detected among sites for all types of disturbance, except fire on large islands but not on small islands.

### 2.5. Population Parameters

**Figure 6:**
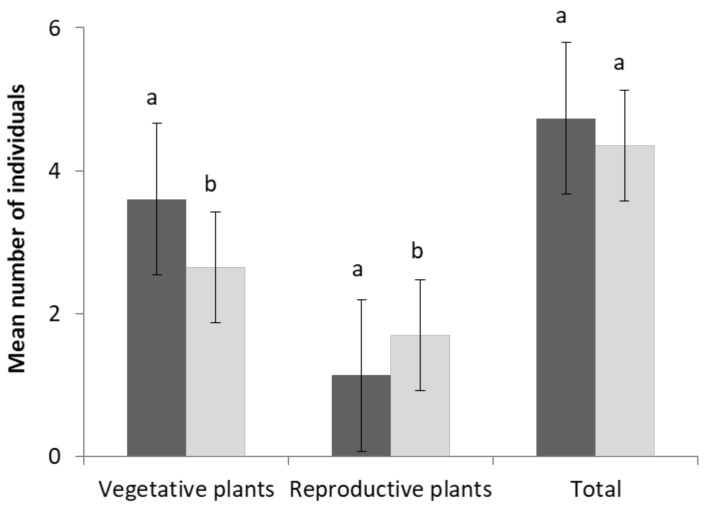
Population structure of *Silene velutina*: mean number of individuals of vegetative plants (seedlings and juveniles), reproductive plants (adults) and total plants per plot on large (dark grey) and small (light grey) Mediterranean islands. A one-way ANOVA was performed to compare the number of individual islands in different age classes; different lowercase letters represent significant differences for each life stage (*p* < 0.05 by SNK test).

The total number of individuals of *S. velutina* was not significantly different (*p* > 0.05) between populations on large and small islands; however, the number of vegetative plants (seedlings and juveniles) was significantly higher on large islands, and the number of reproductive individuals (adults) was significantly higher on small islands (Figure 6). For the size of adult individuals (major axis, minor axis, height), there were no significant differences (*p* > 0.05) between the two groups of islands. Significant differences in various elements of adult size were observed among sites on large islands but not on small islands (data not shown). The reproductive success of adult individuals (the number of floral stems, mature fruits, seeds per fruit and seed set per adult individual) showed no significant differences (*p* > 0.05) in relation to island size. Again, some traits (number of floral stems and seed set per adult individual) showed significant differences (*p* < 0.05) among sites on large but not small islands.

## 3. Discussion

In a literature search prior to our work, we were unable to find examples of studies that had investigated the niche dynamics in plant populations on islands that differ in size. Our study thus provides novel and clear evidence for niche differentiation between populations of *Silene velutina* on large and small islands in the western Mediterranean. In particular, we found significant effects of island size on several parameters related to substrate characteristics (notably the presence/absence of sandy soils and rock cover), elevation, plant cover, community composition and the occurrence of disturbance. We detected a higher variability of environmental conditions on large islands compared to small islands. Our data concord with previous findings that highlight niche differences that are determined by spatial fluctuations in environmental conditions (including disturbance regime) and biological interactions, such as competition [17,38,39]. In addition, other factors not analyzed in this study, such as local adaptation and phenotypic plasticity, may also play a role in niche differentiation at the local level [38]. 

On large islands, we found that variability in substrate composition of populations of *S. velutina* was higher than on small islands, as was the presence of organic matter and species diversity (Shannon index and total number of species) and cover of other species. Habitats on large islands are in a more advanced successional stage (presence of woody vegetation), than on small islands, where woody vegetation is almost absent. In populations on large islands, individuals of *S. velutina* occur in ecological conditions that encompass both sandy and rocky coastal locations, along an environmental stress gradient, from early successional stages close to the sea, to a more inland coastal edge of more mature vegetation (woody communities with *Juniperus* spp.), where environmental stress is lower and resource availability higher, but with more potential competition [40]. On large islands, populations occur in a more heterogeneous array of habitats as a result of disturbance due to grazing, trampling, presence of alien species and rubbish deposition. They also contain a higher proportion of vegetative (younger) individuals, indicative of a life-history strategy dominated by seedling recruitment and turnover. Examples of such a regeneration niche strategy [41] have been reported elsewhere for species and populations in disturbed environments [42]. Indeed, disturbance can create local dynamics of extinction–colonization, in which empty habitat patches are efficiently colonized [18,43]. In particular, disturbance can favor species with high dispersal ability (e.g., high number of juveniles and propagules) and high fecundity [43], but can also produce positive effects for non-competitive species [18].

In contrast, small-island populations of *S. velutina* tend to occur in rocky habitats with less litter cover, the presence of dwarf hemicryptophyte vegetation (ca. 70% of total cover), an absence of phanerophytes and a reduced cover of other species. These populations are characterized by fewer species and an almost complete absence of human-induced disturbance. The ecological niche of populations on small islands is determined by harsh but more homogeneous environments (absence of heterogeneity among sites for 14 of the 22 environmental variables), with low disturbance levels and potentially low competition. A similar type of ecological niche was reported in a comparative study of endemic Mediterranean vascular plants, which were typically found in rocky areas on relatively unfertile substrates, and were associated with stressful habitats with low competition and infrequent human disturbance [34,44].

In addition, although we found no differences in the abundance of individuals in populations on small or large islands, the proportion of reproductive individuals was higher on the former. Small island populations of *S. velutina* may thus have a life-history strategy based more on persistence of adult individuals than on recruitment of juveniles. In particular, the persistence niche strategy [31] is thought to be typical of populations in harsh and/or nutrient-poor environments such as rocky escarpments [45,46], where re-sprouting is prevented by resource limitation, and also in habitats with low levels of disturbance [42].

Populations can reduce or expand their niche breadth when exposed to different disturbance regimes, different competition levels, etc. [39,47]. In particular, niche expansion due to reduced competition (ecological release) is often illustrated for species on islands, where ecological release can be manifested [48,49]. The western Mediterranean endemic *Cyclamen balearicum* Willk. has a narrower ecological amplitude than its more widespread congener *C. repandum* Sm., and its realized niche variability is less among populations in continental France than among populations on the Balearic Islands [44]. Indeed, several endemic species on large Mediterranean islands may show wider niche breadth in relation to reduced competition [21 and references therein].

At the regional scale, we detected a wider niche breadth among *S. velutina* populations on large islands compared to small islands, while at the local scale, the opposite pattern was observed. At the regional scale, the result is probably due to spatial heterogeneity, which is positively correlated with island size, and to the occurrence of disturbance, a factor that can strongly modify species niche breadth due to the production of space that is free from competition [43]. Several studies have reported that generalist species are more frequent in heterogeneous and disturbed environments, while specialist species tend to favor less variable and less disturbed habitats [50,51]. At the local scale, the higher niche breadth of populations on small islands may be due to niche expansion associated with low biodiversity, plant cover and absence of phanerophytes. In the presence of interspecific competition, species can show a space use restriction and a niche shift [39,47]. Consequently, species do not always occupy all their fundamental niche with optimal conditions [52], but only the part in which they are competitively dominant [39]. Similar results have been found for both animals and plants [53,54,55].

Both specialization and niche breadth are conditioned by the methods used for the estimation of niche characteristics [55,56], by the set of considered variables and by the spatial scale at which they are evaluated [28,38]. Consequently, species may be specialist for certain variables on a given spatial scale, but generalist for other variables and on different scales [36,37,55]. However, few studies have considered the effect of spatial scale on niche breadth, and most studies have been based on a few variables that are relatively easy to measure or to detect in spatial datasets [57,58]. Examination of a large number of potential niche axes can, however, help to statistically identify the most important variables and thus better explain observed patterns.

## 4. Materials and Methods

### 4.1. Study Species and Sites

*Silene velutina* belongs to the Western Mediterranean *Silene mollissima* (L.) Pers. aggregate (included in the section Siphonomorpha Otth.), that may be considered an effective example of allopatric speciation of 11 disjunct “schizo-endemic” species [59]. Five of these species (*S. badaroi* Bestr., *S. ichnusae* Brullo, De Marco and De Marco f., *S. hicesiae* Brullo and Signorello, *S. oenotriae* Brullo and *S. velutina* Pourr. ex Loisel.) have a distribution centered on islands in the Tyrrhenian Sea and on the surrounding coastline [60].

*Silene velutina* is endemic to coastal habitats on northeastern Sardinia and southern Corsica and on several adjacent small islands. This chamaephytic species can grow on a variety of rocky, stony and sandy soil substrates derived from both siliceous bedrock degradation and carbonates of organogenic origin [60]. This species has a rosette with floral stems with a maximum height of 80 cm. Flowering is in late spring. Fruits are dehiscent capsules. Seeds tolerate salinity at concentrations similar to seawater and germination can occur until early spring [60]. *Silene velutina* is protected by the 1979 Berne Convention on the Conservation of European Wildlife and Natural Habitats and has a priority status in Annex II of the EU Habitats Directive 43/92/EEC. It is considered vulnerable (VU) in France and endangered (EN) in Italy [61,62,63], but has not been evaluated (NE) in the IUCN International Red Lists [64].

**Figure 7:**
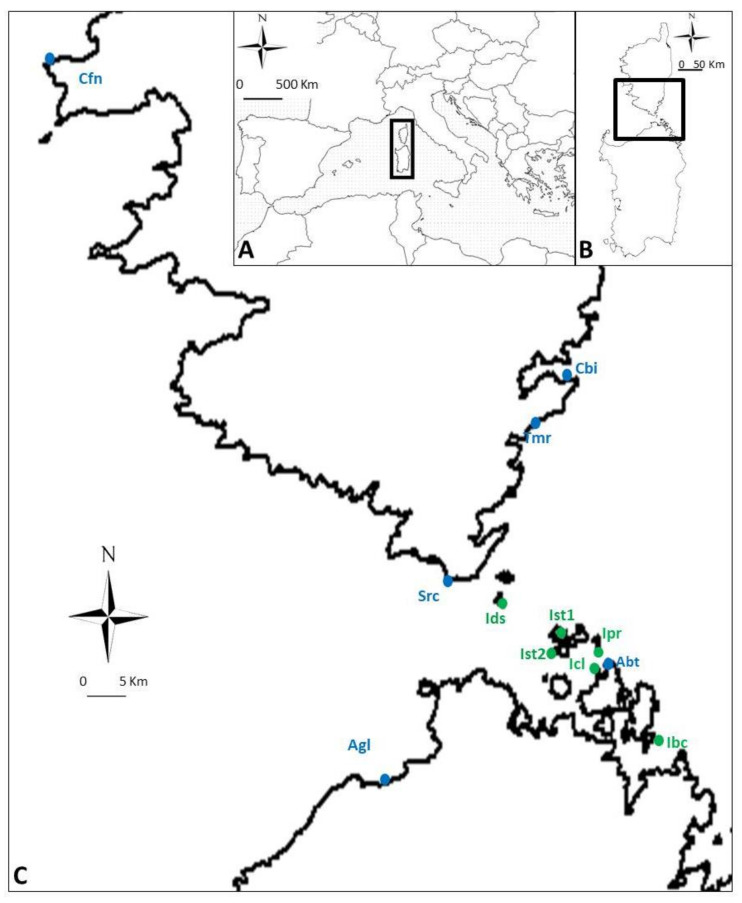
Study area and study sites in the Mediterranean Sea (**A**,**B**). LI (large islands, in blue): Abt—Abbatoggia, La Maddalena; Agl—Riu di Li Saldi, Aglientu; Cbi—Casetta Bianca, Porto Vecchio; Src—Saint Roch, Bonifacio; Cfn—Capu di Fenu, Ajaccio; Tmr—Tamaricciu, Porto Vecchio. SI (small islands, in green): Ibc—Isolotto Baccà, La Maddalena; Ist1—Isolotto Stramanaro 1, La Maddalena; Ist2—Isolotto Stramanaro 2, La Maddalena; Icl—Isolotto Colombo, La Maddalena; Ipr—Isolotto Porro, La Maddalena; Ids—Ilot du Silene, Bonifacio (**C**).

In this study, we sampled populations located on the large islands (LI) of Sardinia, Corsica and La Maddalena (surface area > 49 km^2^) and populations on small islands (SI) that have a surface area < 6 km^2^. Within each group, six sites were randomly selected (Figure 7) from a list of populations [63].

### 4.2. Sampling Design and Response Variables

At each site, within the surface occupied by the species, ten 1 × 2 m plots with a 25 cm grill (45 nodes per plot) were randomly distributed in the population where the plant occurred (following [34,65]).

In each plot, we quantified a range of environmental and population parameters (Table S6). Qualitative variables were quantified in two ways. First, substrate composition in terms of the cover of stones, rocks, sand, soil and litter was assessed as point data for the presence of each element at each node of the plot grid for a total of 5400 point data. Second, drainage and presence of different types of disturbance (grazing, trampling, fire, alien plants, garbage and other unspecified disturbance) were quantified as present or absent in each plot, for a total of 120 replicates. Fire disturbance was derived from public inventories of burnt areas in the last decade. Quantitative variables related to plot localization (elevation, distance from the sea and slope) were noted for each plot (120 replicates). For the study of quantitative biotic variables, the presence of vascular plants was recorded by us at each plot node (5400 replicates) and each species was classified, named and assigned to a particular growth form and chorologic type (also to discriminate alien from native taxa) following Arrigoni [66], Jeanmonod and Gamisans [67] and Pignatti et al. [68]. The plant names follow Euro + Med PlantBase [69] and Bartolucci et al. [70]. Species presence data were used to calculate the total number of species, Shannon and Pielou’s Evenness indices [71,72], the total cover of vascular plants, *S. velutina* cover and other species cover per plot and community composition (number of species for each different life form and coverage of each life form). Moreover, in order to observe whether the community was composed mostly of rare or dominant species, species frequency in one, two or three or more plots was also quantified.

To quantify population structure, all individuals of *S. velutina* in each plot were counted, measured and attributed to one of three life-history stages: (1) *seedlings*: individuals with cotyledons often with one or two pairs of leaves and foliage diameter < 3 cm; (2) *juveniles*: non-reproductive individuals in the year of the study with foliage diameter ≥ 3 cm; (3) *adults:* reproductive individuals in the year of the study. For analyses of population structure, we regrouped seedlings and juveniles as vegetative plants, and adults as reproductive plants. In order to study reproductive success, we noted the number of floral stems for each adult, estimated the number of mature fruits for each floral stem and the number of seeds for each adult plant. Because of the dehiscence of capsules, the number of seeds per fruit was calculated including seed loss, that was estimated for each site by enveloping 6 fruits for 15 individuals in a cotton mesh sachet at the beginning of fruiting. Fieldwork was conducted in July 2014, when all populations were at the same phenological phase (end of fruiting).

### 4.3. Data Analysis

In order to verify whether the frequencies of qualitative variables were related to island size (LI vs. SI), χ^2^ tests were carried out to compare observed values with expected values. Within these analyses, G-tests of heterogeneity were conducted to compare how variables varied among the different sites on each type of island [73]. For the quantitative response variables, including reproductive success, two-way ANOVA (analysis of variance) was used to test the effect of island size (fixed factor) and site (random factor nested within island size). The homogeneity of variances was tested using Cochran’s C-test, and, where necessary, data were appropriately transformed. Student–Newman–Keuls (SNK) tests were carried out to compare mean values when significant differences were detected [74].

Stepwise linear discriminant analysis (LDA) was applied to characterize and discriminate the realized niche in different sites and on the two groups of islands. As an output of the analysis, we obtained a percentage of correct assignment of islands on the basis of their abiotic and biotic features, described above. This method is useful to identify unknown groups characterized both by quantitative and qualitative variables [75]. A combination of predictor variables was found with the aim of minimizing within-group distances and maximizing between group distances to achieve maximum group discrimination [76,77,78]. To reduce overall complexity, differences in the ecological niche of *S. velutina* on large and small islands were visualized by means of principal components analysis (PCA) and graphically represented on three-dimensional axes using the first three discriminant functions. Finally, one-way ANOVA was conducted on the coefficient of variation (CV) to test for an effect of island size on niche breadth at the local and regional spatial scales. On a local scale, the CV was calculated using values of each variable per plot, and on the regional scale the CV was calculated using mean values per site (i.e., mean values of the 10 plots) for groups of variables (e.g., biotic and abiotic variables).

χ^2^ and G-tests of heterogeneity were conducted using an Excel spreadsheet, ANOVA was performed with GMAV5 software package (University of Sydney, Sydney, Australia), and PCA and LDA were carried out by means of SPSS software package release 16.0 (SPSS Inc. for Windows, Chicago, IL, USA).

## 5. Conclusions

In conclusion, our study revealed marked differences in population demographic structures associated with changing environmental parameters at different spatial scales on Mediterranean islands. It provides novel results concerning the ecological niche of the coastal endemic *S. velutina,* particularly in relation to niche differentiation between populations on large and small islands, variations in niche breadth at different spatial scales and changes in demographic structure of island populations. The work clearly shows the need to consider detailed quantitative data, concerning both environmental and population parameters, to correctly characterize the species niche in populations located in different ecological and/or geographical contexts, within the distribution range of each species. Our results confirm the value of obtaining detailed quantitative data concerning both environmental and population parameters on a highly localized scale to correctly characterize the species’ niche in populations located in different ecological and geographic contexts across their distribution range (e.g., [64]). The results provide important information for our understanding of niche differentiation and for the conservation and management of rare endemic species, many of which suffer from a lack of information regarding their ecology and population dynamics.

## Supplementary Materials

The following are available online at https://www.mdpi.com/article/10.3390/plants10112298/s1, Figure S1: Total cover for life forms on LI and SI. Phanerophytes (P), hemicryptophytes (H), chamaephytes (Ch), therophytes (T), geophytes (G), Table S1: LDA: Group classification. Percentages of correct identification after cross-validation, Table S2: LDA: LI sites classification. Percentages of correct identification after cross-validation. Population codes as in Figure 7, Table S3: LDA: SI sites classification. Percentages of correct identification after cross-validation. Population codes as in Figure 7, Table S4: Eigenvectors of the PCA, Table S5: list of vascular plant taxa found at each studied island (BF = Biological Forms). Population codes as in Figure 7, Table S6: Detected environmental and population variables. Response variables that were detected as presence/absence per plot (*n* = 1) and frequency per plot (*n* = 45) are indicated with * and **, respectively. See sampling design for method of sampling.
