# Supplementary material for: Niche Differentiation at Multiple Spatial Scales on Large and Small Mediterranean Islands for the Endemic Silene velutina Pourr. ex Loisel. (Caryophyllaceae)
Source: Plants (Basel). 2021 Oct 26;10(11):2298. doi: 10.3390/plants10112298 (PMC8618681; doi:10.3390/plants10112298)
Supplement: Supplementary file 1 [file plants-10-02298-s001.zip › Table S2.pdf]

Table S2. LDA: LI sites classification. Percentages of correct identification after cross-validation. Population codes as in Fig. 7.

	Abt	Agl	Cbi	Src	Cfn	Tmr	
Abt	**60.0**	10.0	20.0	-	.-	10.0	100.0
Agl	-	**40.0**	-	40.0	20.0	-	100.0
Cbi	10.0	-	**60.0**	-	-	30.0	100.0
Src	-	40.0	-	**60.0**	-	-	100.0
Cfn	10.0	20.0	10.0	.0	**60.0**	-	100.0
Tmr	-	-	10.0	.0	20.0	**70.0**	100.0
