# Supplementary material for: Niche Differentiation at Multiple Spatial Scales on Large and Small Mediterranean Islands for the Endemic Silene velutina Pourr. ex Loisel. (Caryophyllaceae)
Source: Plants (Basel). 2021 Oct 26;10(11):2298. doi: 10.3390/plants10112298 (PMC8618681; doi:10.3390/plants10112298)
Supplement: Supplementary file 1 [file plants-10-02298-s001.zip › Table S3.pdf]

plants

Table S3. LDA: SI sites classification. Percentages of correct identification after cross-validation. Population codes as in Figure 7.

	Ibc	Ist1	Ist2	Icl	Ipr	Ids	Total
Ibc	**20.0**	-	-	10.0	60.0	10.0	100.0
Ist1	60.0	**10.0**	-	30.0	-	-	100.0
Ist2	50.0	10.0	**20.0**	-	-	20.0	100.0
Icl	40.0	10.0	-	**30.0**	-	20.0	100.0
Ipr	70.0	20.0	-	-	**-**	10.0	100.0
Ids	-	30.0	-	-	-	**70.0**	100.0
