# Supplementary material for: Niche Differentiation at Multiple Spatial Scales on Large and Small Mediterranean Islands for the Endemic Silene velutina Pourr. ex Loisel. (Caryophyllaceae)
Source: Plants (Basel). 2021 Oct 26;10(11):2298. doi: 10.3390/plants10112298 (PMC8618681; doi:10.3390/plants10112298)
Supplement: Supplementary file 1 [file plants-10-02298-s001.zip › Table S4.pdf]

Table S4. Factor Analysis—Principal Component Analysis.

**Communalities**		
	Initial	Extraction
Elevation (m)	1.000	.794
Dist. to the sea (m)	1.000	.781
Slope	1.000	.534
Drainage	1.000	.534
% Stone	1.000	.539
% Rock	1.000	.747
% Sand	1.000	.819
% Soil	1.000	.740
% Litter	1.000	.520
Phytophagi	1.000	.526
Greazing	1.000	.396
Trampling	1.000	.286
Fire	1.000	.053
Alien species	1.000	.278
Garbage	1.000	.213
Other disturbances	1.000	.436
Alfa diversity	1.000	.682
Shannon index	1.000	.863
Evenness	1.000	.233
Total coverage %	1.000	.681
Silene coverage %	1.000	.386
Other species coverage %	1.000	.621

Extraction Method: Principal Component Analysis.

**Total Variance Explained**					
Component	Initial Eigenvalues			Extraction Sums of Squared Loadings	
	Total	% of Variance	Cumulative %	Total	% of Variance
1	5.131	23.323	23.323	5.131	23.323
2	4.119	18.722	42.045	4.119	18.722
3	2.412	10.966	53.011	2.412	10.966
4	1.607	7.306	60.317		
5	1.449	6.586	66.903		
6	1.263	5.741	72.644		
7	1.053	4.788	77.433		
8	.918	4.173	81.606		
9	.762	3.462	85.068		
10	.612	2.781	87.849		
11	.574	2.611	90.460		
12	.488	2.218	92.678		
13	.434	1.971	94.648		
14	.327	1.488	96.136		
15	.266	1.208	97.345		
16	.211	.960	98.304		
17	.185	.839	99.143		
18	.077	.350	99.493		
19	.070	.317	99.810		

20	.042	.190	100.000		
21	-8.601E-018	-3.910E-017	100.000		
22	-3.522E-016	-1.601E-015	100.000		

Total Variance Explained

Component	Extraction Sums of Squared Loadings	Rotation Sums of Squared Loadings		
	Cumulative %	Total	% of Variance	Cumulative %
1	23,323	4,650	21,138	21,138
2	42,045	4,264	19,383	40,521
3	53,011	2,748	12,490	53,011
4				
5				
6				
7				
8				
9				
10				
11				
12				
13				
14				
15				
16				
17				
18				
19				
20				
21				
22				

Extraction Method: Principal Component Analysis.

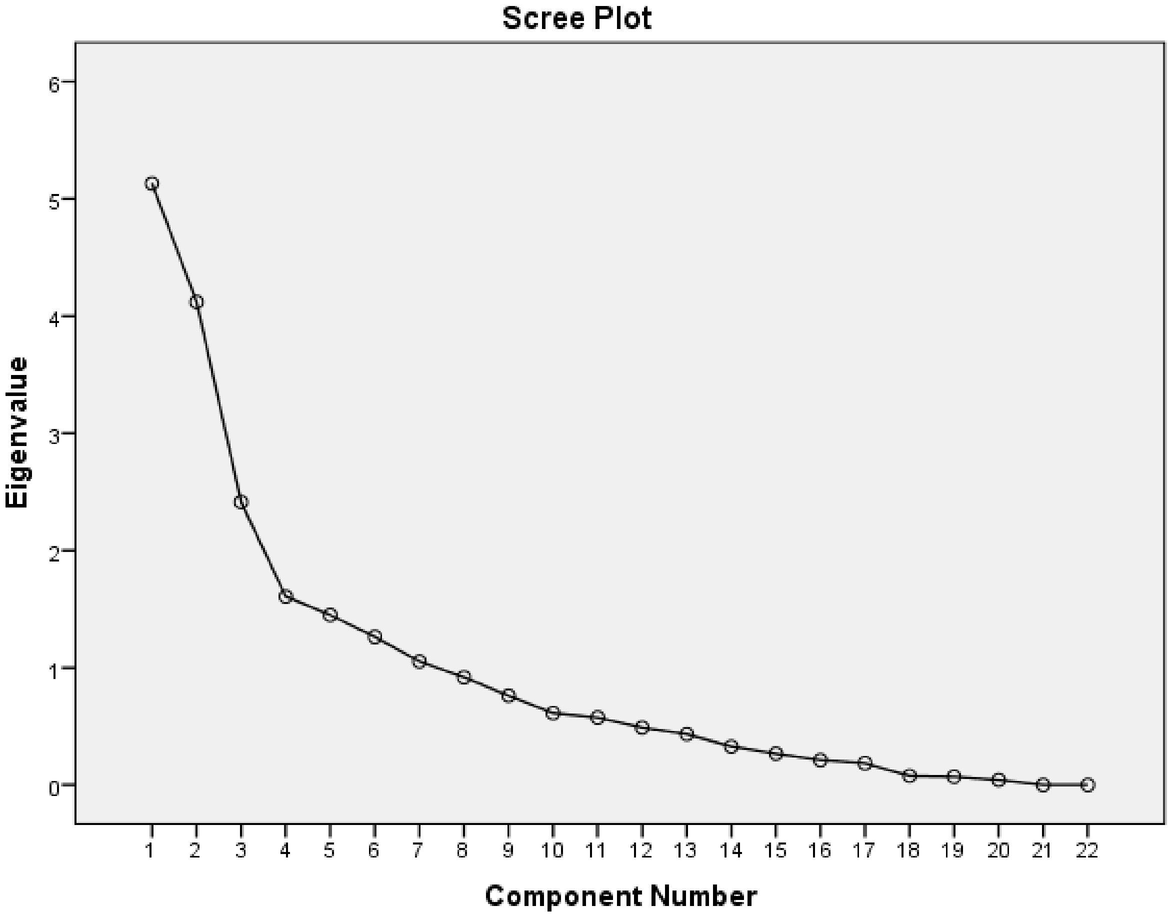

Component Matrix[a]

	Component		
	1	2	3
Elevation (m)	-.006	.888	.069
Dist. to the sea (m)	.084	.880	.004
Slope	-.514	.518	.026
Drainage	-.514	.518	.026
% Stone	-.548	.475	-.110
% Rock	-.856	.066	.104
% Sand	.515	-.320	.673
% Soil	.363	.182	-.758
% Litter	.669	-.238	-.126
Phytophagi	.195	.421	.557
Greazing	-.156	.510	.334
Trampling	.479	-.151	.183
Fire	.190	.023	-.129
Alien species	.260	-.379	.258
Garbage	.432	.020	-.160
Other disturbances	.358	-.198	.518
Alfa diversity	.644	.506	.106
Shannon index	.708	.599	.055
Evenness	.376	.301	-.033
Total coverage %	.687	.219	-.400

Silene coverage %	.046	-.231	-.575
Other species coverage %	.689	.381	-.041

Extraction Method: Principal Component Analysis. [a] Three components extracted.

	Component		
	1	2	3
Elevation (m)	.430	.635	.454
Dist. to the sea (m)	.517	.599	.395
Slope	-.160	.673	.234
Drainage	-.160	.673	.234
% Stone	-.171	.708	.093
% Rock	-.690	.512	.093
% Sand	.065	-.765	.479
% Soil	.602	.181	-.587
% Litter	.457	-.522	-.195
Phytophagi	.217	.007	.692
Greazing	.040	.353	.520
Trampling	.260	-.453	.113
Fire	.202	-.050	-.099
Alien species	-.055	-.520	.073
Garbage	.406	-.183	-.120
Other disturbances	.044	-.531	.389
Alfa diversity	.752	-.038	.340
Shannon index	.866	.010	.337
Evenness	.468	.015	.116
Total coverage %	.783	-.102	-.239
Silene coverage %	.081	-.001	-.616
Other species coverage %	.766	-.107	.154

Extraction Method: Principal Component Analysis. Rotation Method: Varimax with Kaiser Normalization. [a] Rotation converged in 7 iterations.

Component Transformation Matrix

Component	1	2	3
1	.812	-.583	.034
2	.511	.738	.441
3	-.282	-.341	.897

Extraction Method: Principal Component Analysis. Rotation Method: Varimax with Kaiser Normalization.
