# Supplementary material for: Niche Differentiation at Multiple Spatial Scales on Large and Small Mediterranean Islands for the Endemic Silene velutina Pourr. ex Loisel. (Caryophyllaceae)
Source: Plants (Basel). 2021 Oct 26;10(11):2298. doi: 10.3390/plants10112298 (PMC8618681; doi:10.3390/plants10112298)
Supplement: Supplementary file 1 [file plants-10-02298-s001.zip › Table S5.pdf]

plants

MDPI

Table S5. list of vascular plant taxa found at each studied island (BF = Biological Forms). Population codes as in Figure 7.

		LI							SI				
**BF**	**Species**	**Abt**	**Agl**	**Cbi**	**Src**	**Cfn**	**Tmr**	**Ibc**	**Ist1**	**Ist2**	**Ipr**	**Icl**	**Ids**
G	*Allium commutatum* Guss.	x				x			x		x	x	x
H	*Anthemis maritima* L. subsp. *maritima*									x			x
Ch	*Armeria pungens* (Link) Hoffmanns. & Link		x										
P	*Artemisia arborescens* (Vaill.) L.			x	x								
NP	*Asparagus acutifolius* L.				x	x							
NP	*Asparagus albus* L.							x					
P	*Astragalus terraccianoi* Vals.				x								
T	*Atriplex prostrata* Boucher ex DC.									x	x	x	
T	*Avena lusitanica* (Tab.Morais) B.R.Baum		x	x				x					
H	*Bellium bellidioides* L.								x				
T	*Brachypodium distachyon* (L.) P.Beauv.				x								
H	*Brachypodium retusum* (Pers.) P.Beauv.	x	x		x		x		x	x			
T	*Briza minor* L.		x										
G	*Calamagrostis arenaria* (L.) Roth subsp. *arundinacea* (Husn.) Banfi, Galasso & Bartolucci	x	x										
Ch	*Camphorosma monspeliaca* L. subsp. *monspeliaca*				x								
T	*Carlina hispanica* Lam. subsp. *globosa* (Arcang.) Meusel & Kästner		x										
Ch	*Carpobrotus acinaciformis* (L.) L. Bolus	x											
T	*Catapodium pauciflorum* (Merino) Brullo, Giusso, Miniss. & Spamp.		x										
G	*Chamaeiris foetidissima* (L.) Medik.					x							
P	*Cistus salviifolius* L.		x										
G	*Convolvulus soldanella* L.	x											
Ch	*Crithmum maritimum* L.	x	x				x	x					
Ch	*Crucianella maritima* L.		x										
P	*Cytisus laniger* DC.					x							
H	*Dactylis glomerata* L. subsp. *hispanica* (Roth) Nyman							x	x	x			
H	*Daucus carota* L. subsp. *maritimus* (Lam.) Batt.	x			x				x	x	x	x	x
H	*Dittrichia viscosa* (L.) Greuter subsp. *viscosa*				x								
G	*Elymus farctus* (Viv.) Runemark ex Melderis		x	x									
T	*Euphorbia peplis* L.											x	
Ch	*Frankenia laevis* L. subsp. *laevis*				x							x	
Ch	*Helichrysum italicum* (Roth) G.Don subsp. *tyrrhenicum* (Bacch., Brullo & Giusso) Herrando, J.M.Blanco, L.Sáez & Galbany	x	x	x									
T	*Hordeum marinum* Huds.		x										
Ch	*Jacobaea maritima* (L.) Pelser & Meijden subsp. *maritima*		x		x	x				x			x
H	*Juncus acutus* L. subsp. *acutus*	x											
P	*Juniperus phoenicea* L.	x		x	x		x						
T	*Lagurus ovatus* L. subsp. *vestitus* (Messeri) Brullo	x		x	x	x	x						
Ch	*Limonium* sp.				x		x						
P	*Lonicera implexa* Aiton subsp. *implexa*				x								
Ch	*Lotus cytisoides* L.	x		x	x		x	x		x	x	x	x
H	*Malva arborea* (L.) Webb & Berthel.	x			x	x					x	x	
T	*Matthiola tricuspidata* (L.) R.Br.				x		x						
T	*Orobanche sp.*				x	x							
G	*Pancratium maritimum* L.	x											
T	*Parapholis incurva* (L.) C.E.Hubb. subsp. *incurva*	x						x					
P	*Phillyrea angustifolia* L.					x							
P	*Phillyrea latifolia* L.		x										
P	*Pistacia lentiscus* L.		x		x	x	x						
T	*Plantago weldenii* Rchb.						x						
T	*Polypogon subspathaceus* Req.				x			x	x	x	x		x
T	*Portulaca oleracea* L.											x	
P	*Quercus ilex* L. subsp. *ilex*					x	x						
P	*Rubia peregrina* L.		x			x							
Ch	*Ruta chalepensis* L.					x							
T	*Senecio leucanthemifolius* Poir. subsp. *leucanthemifolius*			x									
H	*Silene velutina* Pourr. ex Loisel.	x	x	x	x	x	x	x	x	x	x	x	x
NP	*Smilax aspera* L.		x	x	x	x	x	x					
G	*Umbilicus rupestris* (Salisb.) Dandy					x							
