# Supplementary material for: Niche Differentiation at Multiple Spatial Scales on Large and Small Mediterranean Islands for the Endemic Silene velutina Pourr. ex Loisel. (Caryophyllaceae)
Source: Plants (Basel). 2021 Oct 26;10(11):2298. doi: 10.3390/plants10112298 (PMC8618681; doi:10.3390/plants10112298)
Supplement: Supplementary file 1 [file plants-10-02298-s001.zip › Table S6.pdf]

plants

MDPI

Table S6. - Detected environmental and population variables. Response variables that were detected as presence/absence per plot (N = 1) and frequency per plot (N = 45) are indicated with * and **, respectively. See sampling design for method of sampling.

Variable	Sampling (1X Plot)	Sampling (45 X Plot)
a) ABIOTIC	Elevation (m)<br>Distance from the sea (m)<br>Slope (< 30°; ≥ 30°) *<br>Drainage (Low; Good) *	Stone **<br>Rock **<br>Soil **<br>Sand **<br>Litter **
b) BIOTIC	Alfa diversity<br>Shannon index<br>Evenness index<br>Total coverage<br>*Silene velutina* coverage<br>Other species coverage<br>Number of species for life form<br>Coverage for life form	Species (vascular plants)<br>Life form of species
c) DISTURBANCE	Phytophagi and parasites *<br>Grazing *<br>Trampling *<br>Fire *<br>Alien species *<br>Garbage *<br>Other disturbances *	
d) POPULATION	Abundance of individuals (seedlings, saplings, adults, total)<br>Adult size (major axis, minor axis, height)<br>Number of floral stems x adult<br>Number of mature fruits x floral stem<br>Number of seeds x fruit<br>Seed set x adult	
