# Supplementary material for: Niche Differentiation at Multiple Spatial Scales on Large and Small Mediterranean Islands for the Endemic Silene velutina Pourr. ex Loisel. (Caryophyllaceae)
Source: Plants (Basel). 2021 Oct 26;10(11):2298. doi: 10.3390/plants10112298 (PMC8618681; doi:10.3390/plants10112298)
Supplement: Supplementary file 1 [file plants-10-02298-s001.zip › Figure S1.pdf]

plants

Appendix S1

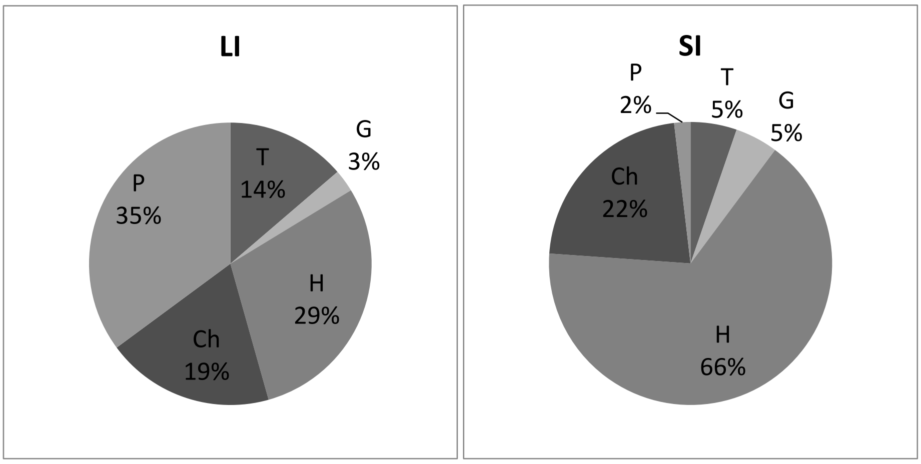

Figure S1.1 - Total cover for life forms on LI and SI. Phanerophytes (P), nano-panerophytes (NP) hemicryptophytes (H), chamaephytes (Ch), therophytes (T), geophytes (G), hydrophytes (I).
