# Supplementary material for: Niche Differentiation at Multiple Spatial Scales on Large and Small Mediterranean Islands for the Endemic Silene velutina Pourr. ex Loisel. (Caryophyllaceae)
Source: Plants (Basel). 2021 Oct 26;10(11):2298. doi: 10.3390/plants10112298 (PMC8618681; doi:10.3390/plants10112298)
Supplement: Supplementary file 1 [file plants-10-02298-s001.zip › Table S1.pdf]

plants

Table S1. LDA: Group classification. Percentages of correct identification after cross-validation.

	LI	SI	Total
LI	**86.7**	13.3	100.0
SI	3.4	**96.6**	100.0
